# Hydroxypropyl methylcellulose-induced urticaria and angioedema: a case report

**DOI:** 10.1186/s13223-026-01021-5

**Published:** 2026-03-06

**Authors:** Won Jin Lee, Eun-Jung Jo, Hye-Kyung Park

**Affiliations:** 1 Division of Allergy and Clinical Immunology, Department of Internal Medicine, Pusan National University Hospital, Busan, Korea; 2 Department of Internal Medicine, Pusan National University School of Medicine, 179 Gudeok-ro, Seo-gu, Busan, 49241 Korea

**Keywords:** Hydroxypropyl methylcellulose, Urticaria, Angioedema, Hypersensitivity, Excipients

## Abstract

**Background:**

Hydroxypropyl methylcellulose (HPMC) is a food additive widely used as an excipient in oral pharmaceutical formulations. While widely used in oral formulations, its potential as an ingestible allergen remains largely unrecognized.

**Case presentation:**

A 47-year-old man developed urticaria and angioedema after ingesting an HPMC-containing product, including a dietary probiotic drink and a digestive enzyme tablet. He presented with periorbital swelling and generalized urticaria within one hour after consuming a dietary probiotic drink. Two months prior, the patient experienced generalized urticaria and facial swelling after concomitant intake of a vitamin C drink and a digestive enzyme tablet. Skin prick tests revealed positive reactions to the capsule shell of the probiotic drink and digestive enzyme tablet. HPMC was identified as a common excipient in both digestive enzyme tablet and the capsule shell of dietary probiotic drinks and intradermal tests confirmed hypersensitivity to HPMC. An oral provocation test with HPMC induced urticaria, further supporting the diagnosis.

**Conclusions:**

This case demonstrates that HPMC can induce immediate hypersensitivity reactions via the oral route. HPMC should be considered a potential culprit in patients with unexplained recurrent allergic reactions to oral medications or dietary supplements, particularly when multiple formulations are involved.

## Background

Food additives are substances intentionally added to food during its production, processing, packaging, transportation, or storage to improve stability, texture, appearance, and shelf life [1]. Food additives are categorized into various groups, including preservatives, colorants, flavor enhancers, sweeteners, stabilizers, and emulsifiers [2]. Commonly recognized additives include annatto, aspartame, erythritol, gelatin, monosodium glutamate, nitrates, polyethylene glycol, and polysorbate [3].

Although adverse reactions to food additives are generally considered uncommon, the prevalence of such reactions remains uncertain and has been estimated to be approximately 1% in the general population [3]. Importantly, food additive allergies are frequently underrecognized in clinical practice, as nonspecific symptoms and ubiquitous exposure often mask the underlying cause. Consequently, these reactions may be overlooked unless meticulous ingredient histories and targeted diagnostic evaluations are performed [4].

Hydroxypropyl methylcellulose (HPMC) is a food additive registered in the International Numbering System as No. 464 and designated by the Codex Alimentarius Commission [1]. HPMC is widely used as an emulsifier, thickener, or stabilizer in processed foods and is also extensively incorporated into pharmaceutical formulations, where it plays a central role in modified-release oral preparations. In addition, HPMC is commonly used as a constituent of capsule shells intended for oral, inhalational, and rectal administration [5].

Despite its widespread use and long-standing reputation for safety, hypersensitivity reactions to HPMC have rarely been reported. To date, only one case of anaphylaxis occurring during cataract surgery following exposure to an HPMC-containing ophthalmic preparation has been described [6]. Although the limited number of published reports may reflect a low frequency of HPMC hypersensitivity; underrecognition of HPMC as a potential culprit in the evaluation of suspected food- or drug-related allergic reactions cannot be excluded. Here, we report a case of HPMC hypersensitivity presenting with urticaria and angioedema, highlighting HPMC as a potential hidden allergen in the evaluation of unexplained immediate hypersensitivity reactions.

## Case presentation

A 47-year-old man developed urticaria and angioedema, including periorbital edema, within one hour after consuming a probiotic drink one month prior to presentation. The probiotic product was a single commercially available preparation consisting of two separately packaged components-a liquid formulation and an orally administered capsule-that were intended to be taken together. Two months previously, he experienced generalized urticaria and edema following consumption of a vitamin C beverage and a digestive enzyme tablet. On both occasions, the symptoms improved with oral antihistamine treatment alone and resolved within several hours.

The patient had a history of hyperlipidemia, prediabetes, chronic spontaneous urticaria, allergic rhinitis, and hypersensitivity to nonsteroidal anti-inflammatory drugs and had experienced an allergic reaction to iodinated contrast media. The patient was taking medication for benign prostatic hyperplasia (BPH).

At the time of the visit, the symptoms had resolved. We investigated the ingredients of the substances consumed during the previous episodes (Table 1). HPMC was identified as a common excipient in both the capsule shell of the probiotic drink and the digestive enzyme tablet, while microcrystalline cellulose, stearic acid magnesium, and silicon dioxide were identified as common excipients in both the capsule component and the digestive enzyme tablet. In contrast BPH medications, which the patient had been taking without any adverse reactions, contained no such excipients. A skin prick test (SPT) was performed using the liquid component of the probiotic drink, dissolved capsule components, capsule shell, and digestive enzyme tablet. The criterion for a positive reaction was a wheal ≥ 3 mm in size or equivalence to the positive control solution after 15 min, accompanied by erythema. SPTs showed positive results for both the capsule shell and digestive enzyme tablet. Consequently, additional skin and oral provocation tests were performed to investigate HPMC allergy as it was a common component of both.

**Table 1:** Major ingredients and excipients of substances administered to the patient

	Digestive enzyme tablet	Vitamin C drink	Probiotic drink	
Major ingredient	Cellulase pancreatin simethicone	Vitamin C fructose	*Lactobacillus*	
Excipient list	Microcrystalline cellulose	Synthetic flavor	Capsule component	*L. courvatus* HY7601
	Tablet shield green	Trisodium citrate		*L. plantarum* KY1032
	Stearic acid magnesium	Enzyme-treated stevia		Lactose
	Silcon dioxide	Pectin		Microcrystalline cellulose
	Polyethylene glycol 6000			Silcon dioxide
	Sodium starch glycolate			Stearic acid magnesium
	Hypromellose		Capsule shell	Gelatin
	Hydroxypropyl methylcellulose			Phthalate hydroxypropyl methylcellulose
	Lactose monohydrate			Hydroxypropyl methylcellulose
	Croscarmellose sodium			Glycerin fatty acid ester
				Purified water
				Titanium dioxide
				Sucrose fatty acid ester
			Probiotic drink (liquid)	Indigestible maltodextrin
				Purified water
				Fructooligosaccharide
				Xylitol
				Red beet concentrate
				Lemon concentrate
				Citric acid
				Red grapefruit concentrate
				7 Vegetable mixture
				Vegetable juice mixture
				Fish collagen peptide
				Flavor
				Enzyme-treated stevia
				Flavor (grapefruit, kale)
				Hyaluronic acid
				Elastin powder
				Milk, soy, beef, pork

Since a non-irritating concentration of HPMC has not been established, we used a 2% HPMC solution, per a previous report [6]. For the SPT, we prepared dilutions of 1, 1/10, and 1/100 HPMC, and for the intradermal test (IDT) a 0.2% HPMC solution was used as the base from which dilutions of 1, 1/10, and 1/100 were prepared. The HPMC oral provocation test was performed with a cumulative dose of 8 g.

The IDT was positive for HPMC up to a dilution of 1/100 (Table 2). In contrast, two control participants showed negative HPMC SPT results. The HPMC oral provocation test revealed wheals in the arms and legs within 1 h after reaching the final cumulative dose of 8 g. The patient was diagnosed with an HPMC allergy and instructed to avoid HPMC-containing medications. A drug safety card was also provided. The patient and two control participants provided informed consent, and the report was approved by the Institutional Review Board of Pusan National University Hospital (IRB No. 2412-016-146).

**Table 2:** Results of skin prick test and intradermal test

	Saline (SPT)		Histamine(SPT)		Digestive enzyme tablet (SPT)		Capsule shell of probiotic drink (SPT)		Capsule component of probiotic drink (SPT)		Probiotic drink (SPT)		HPMC (SPT)		HPMC (IDT)	
	Wheal (mm × mm)	Flare (mm × mm)	Wheal (mm × mm)	Flare (mm × mm)	Wheal (mm × mm)	Flare (mm × mm)	Wheal (mm × mm)	Flare (mm × mm)	Wheal (mm × mm)	Flare (mm × mm)	Wheal (mm × mm)	Flare (mm × mm)	Wheal (mm × mm)	Flare (mm × mm)	Wheal (mm × mm)	Flare (mm × mm)
1/100	NT	NT	NT	NT	4 × 7	22 × 20	3 × 3	17 × 15	–	–	–	–	–	–	5 × 5	13 × 10
1/10	NT	NT	NT	NT	5 × 8	27 × 25	4 × 6	19 × 22	–	–	–	–	–	–	10 × 10	27 × 25
1	-	-	4 × 4	17 × 20	8 × 10	38 × 32	7 × 7	32 × 30	–	–	–	–	–	–	13 × 10	28 × 30

HPMC, hydroxypropyl methylcellulose; SPT, skin prick test; IDT, intradermal test; NT, not tested

### Discussion and conclusions

To the best of our knowledge, this is the first reported case of an immediate hypersensitivity reaction to hydroxypropyl methylcellulose (HPMC) occurring specifically after oral ingestion. In contrast to its widespread application, the only previously reported case of HPMC hypersensitivity involved anaphylaxis after topical ophthalmic use, which was definitively confirmed by skin prick testing [6]. Although reports of HPMC hypersensitivity are extremely rare, HPMC allergy should be considered, particularly in patients in whom drug allergy is suspected but cannot be attributed to a specific active ingredient, as in the present case. Food additive and excipient allergies are easily overlooked if individual components are not carefully reviewed, and their diagnosis remains challenging owing to the limited understanding of the underlying immunopathogenic mechanisms.

HPMC, one of the most commonly used cellulose ethers [5], is widely incorporated into food products, pharmaceuticals, and cosmetics and is generally considered safe. However, as demonstrated in the present case, it can trigger allergic reactions in susceptible individuals. HPMC and carboxymethylcellulose (CMC) are structurally related cellulose-derived excipients that share a common β-1,4-linked polysaccharide backbone and differ mainly in their chemical substitutions [7]. While hypersensitivity to HPMC is exceedingly rare, immediate hypersensitivity reactions to CMC have been increasingly reported, including urticaria, angioedema, and anaphylaxis, most commonly after parenteral exposure [8–10]. These observations indicate that cellulose-based excipients, traditionally regarded as inert, can act as clinically relevant allergens.

The shared cellulose backbone between HPMC and CMC raises the theoretical possibility of immunologic cross-reactivity. Recently, a case reporting potential cross-reactivity between CMC and HPMC has been described [11] providing emerging but still limited clinical evidence for cross-reactivity among cellulose-based excipients. However, current data are confined to single-patient observations, and systematic evidence supporting consistent or predictable cross-reactivity between HPMC and CMC is lacking. Therefore, it remains unclear whether sensitization is directed against shared polysaccharide structures of the cellulose backbone or against specific chemical substitutions unique to each derivative.

In addition, previous reports of CMC hypersensitivity have suggested that clinical reactivity may depend on the route of exposure. Some patients developed immediate allergic reactions to CMC after parenteral or topical administration, while tolerating CMC-containing products administered via other routes, including oral exposure [12].

These observations indicate that tolerance to an excipient through one route does not necessarily exclude clinically relevant hypersensitivity when the same excipient is administered via a different route [12]. Given that HPMC is a structurally related cellulose derivative, a similar route-dependent pattern may be clinically relevant. The previously reported anaphylaxis case occurred after ophthalmic exposure, whereas the present case followed oral ingestion, underscoring the importance of considering the route of exposure when evaluating suspected hypersensitivity to cellulose-derived excipients.

This case report has several limitations. First, data regarding the appropriate HPMC concentration for skin testing are lacking. However, based on previous reports, we confirmed a negative response in two control subjects to ensure a non-irritating concentration. Second, there is no established criteria for provocation testing with HPMC. Because HPMC is used as an excipient rather than a therapeutic agent, the concept of a therapeutic dose cannot be defined. The Joint FAO/WHO Expert Committee on Food Additives (JECFA) has designated the acceptable daily intake of HPMC as “not specified”. Laxative effects have been reported as known adverse effects of HPMC. JECFA (1990) stated that a total dietary fiber intake of up to 30 g/day, including modified cellulose, is considered safe. Accordingly, we conducted an oral provocation test in a stepwise manner with a cumulative dose of 8 g [13]. Third, serum tryptase was not measured because the patient presented after complete resolution of symptoms and showed no hypotension or evidence of systemic organ involvement. Although tryptase measurement might have provided additional supportive information, its absence represents a limitation of this report. In addition, while an IgE-mediated mechanism is suspected based on the clinical presentation and positive skin test results, this could not be definitively confirmed. Finally, quantitative information on HPMC content in different formulations was unavailable despite contacting manufacturers, precluding dose–response assessment. No prior exposure to HPMC-containing medications was identified in the patient’s medication history, and the potential influence of the route of exposure on the severity of HPMC hypersensitivity could not be evaluated.

In conclusion, this case provides clinical evidence that HPMC can trigger immediate hypersensitivity reactions following oral ingestion, a route previously unrecognized for this allergen. Further research is essential to elucidate the immunological mechanisms underlying these reactions to cellulose-based additives. Moreover, the establishment of standardized, non-irritating concentrations and validated diagnostic protocols for HPMC is necessary to facilitate accurate diagnosis and management. Increasing clinical awareness of excipient-related allergies among healthcare providers will be essential to prevent overlooked reactions and to ultimately improve patient safety in both dietary and pharmaceutical settings.

## Data Availability

This article contains all data generated or analyzed during this study.

## Abbreviations

HPMC: Hydroxypropyl methylcellulose
BPH: Benign prostatic hyperplasia
SPT: Skin prick test
IDT: Intradermal test
CMC: Carboxymethylcellulose
JECFA: Joint FAO/WHO expert committee on food additives
